# Implementing a Virtual, Collaborative Care Weight Management Program in Rural Primary Care: Pilot Results and Insights

**DOI:** 10.1177/26924366251385717

**Published:** 2025-10-20

**Authors:** Sarah Hales, Caitlin Koob, Jillian Harvey, Ryan Kruis, James McElligott, Dee Ford

**Affiliations:** ^1^ Center for Telehealth, Medical University of South Carolina, Charleston, South Carolina, USA.; ^2^ Department of Psychiatry and Behavioral Sciences, College of Medicine, Medical University of South Carolina, Charleston, South Carolina, USA.; ^3^ Department of Healthcare Leadership and Management, Medical University of South Carolina, Charleston, South Carolina, USA.; ^4^ Manatt Health Strategies, Manatt, Phelps & Phillips, LLP, Chicago, Illinois, USA.; ^5^ Department of Pediatrics, Medical University of South Carolina, Charleston, South Carolina, USA.; ^6^ Division of Pulmonary and Critical Care Medicine, Medical University of South Carolina, Charleston, South Carolina, USA..

**Keywords:** collaborative care model, obesity, primary care, telehealth, weight management

## Abstract

**Background:**

Obesity rates have reached epidemic levels globally, and still, rates of overweight and obesity in South Carolina (SC) remain disproportionately high compared with national statistics. Further, those living in rural areas are at an increased risk of experiencing obesity with limited access to weight management programs, such as specialized nutrition counseling and lifestyle behavior change programs. Collaborative care management (CoCM) is an evidence-based approach that leverage a multidisciplinary team to treat specialized conditions within primary care, most often implemented to manage mental and behavioral health conditions. To our knowledge, the Primary Care Integrated Weight Management program (PCIWM) is the first to adapt CoCM model for weight management.

The purpose of this study is to (1) describe pilot PCIWM implementation and (2) identify strategies to improve adoption and inform sustainability.

**Methods:**

A multidisciplinary care team—including Registered Dietitians Nutritionists (RDNs), primary care providers (PCPs), practice managers, and a weight management consultant—was established to develop a PCIWM workflow and patient registry within an academic medical center and four affiliated rural primary care clinics. The PCIWM workflow involved RDN documentation, routing encounter notes to PCPs, monthly consultations between RDNs and weight management consultants, and between-session surveys.

Adult patients with weight-related concerns, particularly those with a body mass index (BMI) >25 kg/m^2^, were referred to PCIWM. Participants underwent a nutrition assessment, set goals, received medical nutrition therapy from RDNs, integrated within their care plans, and were offered monthly RDN sessions. Data were sourced from electronic health records, PCIWM encounters, and survey responses. Descriptive statistics were used to examine baseline patient characteristics, initial service utilization from PCIWM, and survey responses.

**Results:**

Participants’ mean age was 46.8 years, a majority were female (85.3%) identified as White (65.5%), mean baseline weight was 115.0 kg, and mean baseline BMI was 41.82 (SD = 11.4, [22.25, 78.85]) (*N* = 61). 29% were actively engaged and had a mean of 3.4 RDN visits (mean duration = 100.7 min). The majority of survey respondents (*N* = 11, 63.6%) reported adhering to their goals. Additionally, 51.9% (*n* = 14 of 27) of participants who attended their scheduled appointments and had updated weights maintained (within 1 kg) or lost weight at time of follow-up. Patients’ demonstrated mean BMI reductions of 2.7%.

**Conclusions:**

PCIWM provides a unique model for virtual weight management, and pilot data suggest PCIWM is a feasible approach. Still, further research and advocacy efforts are needed for supportive policy and program expansion to manage patient’s weight, cardiovascular risk, and other related health outcomes.

## Introduction

Obesity rates have reached epidemic levels globally, and still, rates of overweight and obesity in South Carolina (SC) remain disproportionately high compared with national statistics.^1–4^ The associations between diagnosed obesity and a myriad of negative health outcomes are well-documented, including type II diabetes mellitus, cancer, and cardiovascular conditions.^5–8^ Further, those living in rural areas are at an increased risk of experiencing obesity with limited access to weight management programs, such as specialized nutrition counseling and lifestyle behavior change programs.^9–13^ Barriers, including limited health care infrastructure, required travel, shortage of specialty providers, and cost, often hinder access to necessary health care services in rural areas.^12,14,15^ Despite lack of access to specialty care in rural settings, patients typically have access to primary care providers (PCPs) in their community. Obesity is an increasing concern in primary care with clinical guidelines reflecting this priority area^16^; however, PCPs report significant barriers (i.e., lack of time, knowledge, training) to effectively treat obesity and associated chronic diseases in the primary care setting.^17^ Despite these aforementioned challenges, developing models of team-based care was identified as an opportunity to improve obesity treatment in the primary care setting.^17^

Evidence suggests telehealth, defined as the provision of health care services via internet-connected devices, may override barriers to care and effectively improve access to specialized health care services among those residing in rural SC.^9,18–20^ Through telehealth, medical providers can virtually provide an array of specialty services including psychiatry, nutrition, and other medical subspecialties.^19–22^ Prior studies have shown promise in addressing weight management concerns among telehealth-delivered interventions, citing improved patient engagement and significant weight loss in some cases.^9,23,24^ A virtual, telephone-based counseling program found patients had reduced weight regain and increased the proportion of long-term weight reduction of at least 10%, with extended participation to 22 months.^25^ Additional research suggests telehealth weight management programs may provide necessary support for patients to remain engaged and benefit from reduced risk of cardiovascular-related other chronic diseases, and improved function and decreased pain among those with orthopedic conditions.^26–28^ Still, there is a wide variety in the delivery of telehealth interventions for weight management, often tailored for specific populations with variable check-in schedules, intervention methods and measures, and length of follow-up periods.^12,23,27,28^

Within this health system, nutrition consultation with a Registered Dietitian Nutritionist (RDN) was among the most sought-after subspecialty from 2012 through 2017,^20^ and subsequently established into its own service line of outpatient nutrition services, provided via telehealth (termed “telenutrition”). An evaluation of this telenutrition service demonstrated robust utilization even after the transition to direct-to-consumer virtual visits during COVID-19; however, lack of insurance coverage and lack of treatment plan integration between the RDNs and referring providers were prominent program limitations.^13^

In an effort to optimize our telenutrition service and assist PCPs in addressing obesity in primary care, a multidisciplinary team was formed to develop a new model of obesity care, based on the collaborative care model (CoCM) for behavioral health. CoCMs are an evidence-based approach that leverage a multi-disciplinary team to treat specialized conditions within primary care, and have been implemented among patients with comorbid depression and obesity with demonstrated improved health outcomes and increased health care access.^29–32^ Therefore, building from documented evidence in telehealth and CoCM,^9,29–31^ the Primary Care Integrated Weight Management program (PCIWM) was developed to offer an innovative, virtual care model for nutrition counseling and weight management among adult patients living in rural SC. The purpose of this study was to develop PCIWM and evaluate its pilot implementation among adult patients who received care at rural primary care clinics affiliated with a large academic medical center in SC. To our knowledge, this is the first program to adapt CoCM for weight management. Therefore, the aims of this study are to (1) describe the pilot implementation of PCIWM and (2) identify strategies to improve adoption and inform optimization and sustainability.

## Materials and Methods

This study took place at an academic health center and four of its affiliated rural primary care clinics in SC. Using CoCM as a guide,^29–31^ a multidisciplinary care team was established to develop a new workflow and patient registry for this novel PCIWM program. This study was deemed a quality improvement project or program evaluation from the Medical University of South Carolina’s Institutional Review Board and did not require further review.

### Program description

PCIWM was developed to increase access to nutrition and weight management counseling in primary care and to treat obesity in rural primary care clinics via telehealth throughout SC.^9,19^ The PCIWM team from the academic medical center included the medical director of telehealth, the nutrition services manager, the weight management consultant, the coordinator for outpatient telehealth services, two adult RDNs, the practice manager(s), and the eight PCPs at these rural clinics (Table 1).

**Table 1.** PCIWM Team Roles and Responsibilities

Team member	Roles/responsibilities
Academic health center	
Medical director, Center for Telehealth	• Develop initial pilot program concept
	• Oversee overall program development, implementation and evaluation
Service champion for outpatient telehealth nutrition services	• Planning and development of pilot program team, workflow, and implementation and evaluation
	• Attended quarterly team planning meetings
Coordinator for outpatient telehealth services	• Coordination of all outpatient telehealth services, including PCIWM
	• Liaison between primary care practice team and academic health center team
	• Attended quarterly planning meetings
Registered dietitians nutritionists	• Provide initial dietary assessment of patients referred to outpatient telehealth nutrition services
	• Establish weight management goals with patients
	• Provide monthly medical nutrition therapy with patients
	• Document patient notes and route treatment plan updates to PCPs via Epic
	• Between-session contact with patients
	• Maintain the PCIWM patient registry
	• Attend monthly consultation meetings with weight management consultant to adjust patient goals as necessary
Weight management consultant (PhD)	• Attend monthly consultation meetings with RDNs and oversee treatment goals, and make recommendations for those patients not meeting their goals
MUSC's Center of Excellence team (i.e., research and evaluation support) team	• Epic data abstraction, synthesis, and reporting
Affiliated primary care practices	
Primary care practice manager	• Liaison between primary care practice staff and care team and the academic health center team
	• Attend quarterly planning meetings
Primary care providers (e.g., Medical doctor, nurse practitioner)	• Provide medical intervention to their patients at primary care visits
	• Check-in with patients regarding weight management goal attainment
	• Prescribe medication to treat medical conditions, including obesity
	• Order necessary laboratories and communicate results to patients
	• Follow-up with RDNs regarding questions or concerns with weight management treatment plan/goals
Patients	• Participate in weight management treatment plan development
	• Participate in behavior change to achieve weight management goals
	• Attend primary care visits with PCP
	• Attend RDN appointments
	• Complete between session check-ins from RDN

PCP, primary care providers; PCIWM, Primary Care Integrated Weight Management program; RDN, Registered Dietitians Nutritionists.

### PCIWM program workflow

**FIG. 1.**
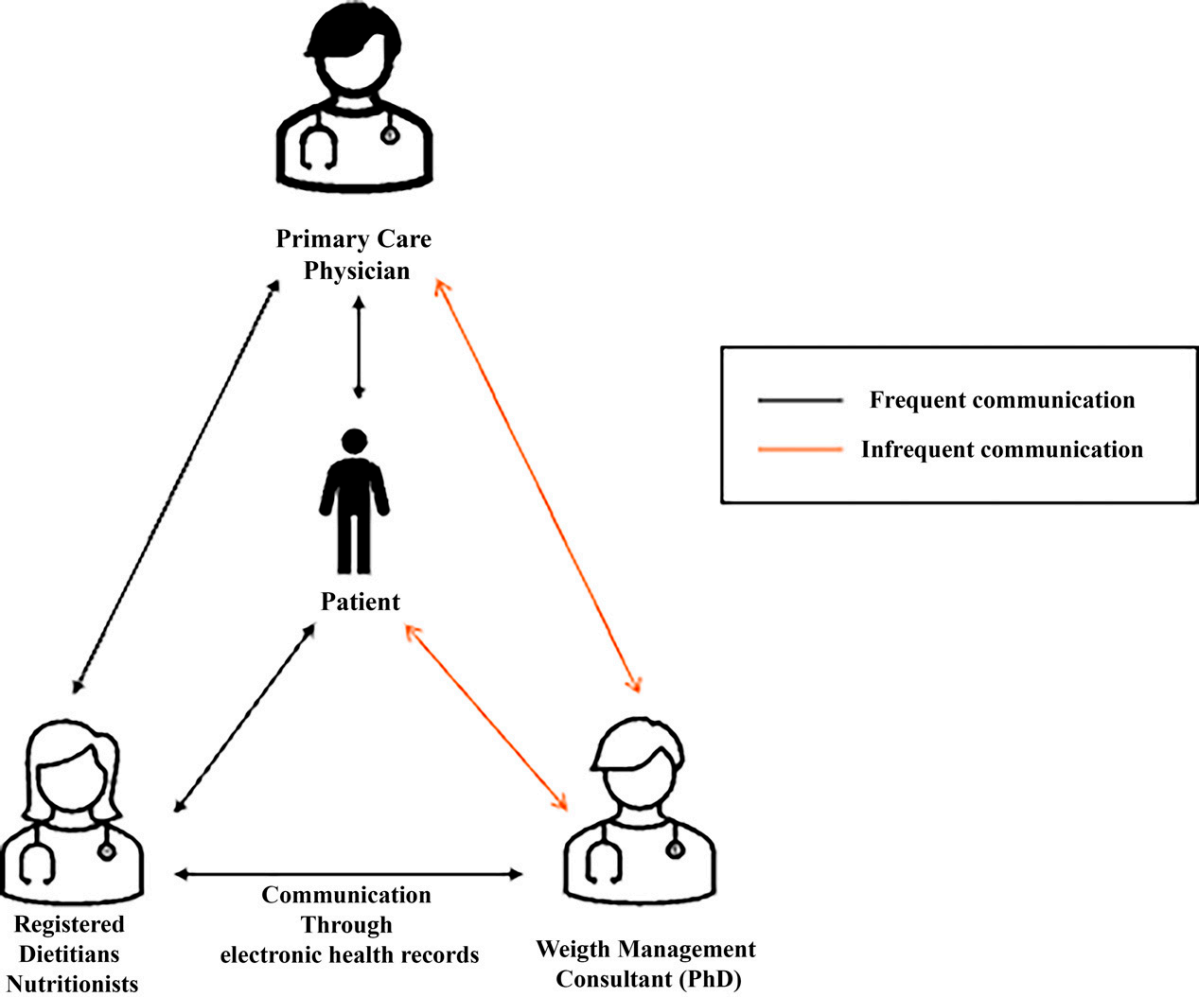
The collaborative care model for behavioral health, adapted for weight management.^40^

While PCIWM was developed as an extension of pre-existing telenutrition services provided by this health system, subsequent adaptations were involved to finalize the PCIWM workflow.^13,20^ The resulting PCIWM workflow (Fig. 1) comprised charting of RDN encounters with patients, electronic routing of encounter notes to PCPs with a brief synopsis of the current treatment plan highlighted in the comments section of the routed note, a separate patient registry maintained by the RDNs, and monthly consultations between RDNs and the weight management consultant. At a patient level, referrals for nutrition counseling with an RDN were made due to patients’ body mass index (BMI) status within the overweight or obese range—with or without other comorbidities (i.e., hypertension, hypercholesterolemia, sleep apnea). Once patients were referred to telenutrition services, the RDN confirmed that the patient’s goals included weight loss and that the patient was not receiving contradictory services (i.e., kidney failure, eating disorder). Then, the RDN added the patient to the PCIWM registry, proceeded with connection to RDN services, and provided ongoing care. At the clinic level, quarterly meetings were conducted with the telehealth service champion, the telehealth coordinator, and the practice manager to check-in regarding referral volume and source(s), share updates, and troubleshoot practice-level concerns (Fig. 1).

During quarterly meetings, clinicians also identified patients who may warrant escalation in weight management care. If escalation was warranted, particularly among patients who were not progressing or achieving their goals despite patient-reported best efforts, clinicians collaboratively discussed alternative referral and treatment options. Recommendations from RDNs and weight management consultants for a more structured, staged approach to care were routed to PCPs for follow-up at the patient’s next appointment. For instance, adjustments to patients’ care plans may require medical monitoring or may require transition from monthly to weekly sessions. Conversely, patients without escalation needs continued the program as intended.

Additionally, an asynchronous online survey was developed by the RDNs and a weight management consultant and emailed to patients between virtual one-to-one sessions with the RDN to check-in and assess when escalation of care was warranted. Surveys were distributed once, regardless of responsiveness. Survey responses were stored in RedCap, an electronic database that is compliant with the Health Insurance Portability and Accountability Act.^33^

### Patient inclusion/exclusion criteria

At baseline, patients who were at least 18 years old and had a BMI above 25 kg/m^2^ were eligible to participate in PCIWM. Patients were not eligible to participate if they were currently pregnant and/or had a diagnosis of type 1 diabetes mellitus, an eating disorder, end-stage renal disease, or malnutrition. Of note, one patient actively sought treatment for consistent weight gain and was enrolled as an exception, regardless of BMI below the intended range (BMI = 22.25).

Upon participating in the program, patients’ level of engagement was categorized as active, inactive, or withdrawn. “Active” engagement was defined as patients who had at least one interaction with the RDN within a 3-month period. Further, patients were considered to be “inactive” but still participating in the program if they had zero engagement with an RDN for up to 3 months, and patients were progressed to “withdrawn” after having no engagement with an RDN—despite clinician’s attempts to re-engage—in more than 3 months.

In addition, patient’s “progress” was based on clinical assessment and reasoning on a case-by-case basis. For instance, despite a patient’s weight maintenance and/or change, they would be considered “progressing” if they were achieving goals related to diet quality or portion control. However, patients who were not “progressing” over the 3-month period may be considered withdrawn and transferred to another program or intervention by the interdisciplinary team. If a patient, the RDN, or the weight management consultant felt the patient could benefit from a different type of intervention, then a recommendation was made to the PCP for a referral to a specific type of program (i.e., medication, bariatrics, or a structured lifestyle change program for weight loss). Of note, patients could elect to withdraw from the program at any time without question.

### Dietary intervention

Patients received medical nutrition therapy from the RDNs as the dietary intervention within PCIWM. As part of the intervention, each patient completed a full nutrition assessment and established individual nutrition-related goals with the RDN. Then, patients were offered monthly follow-up sessions for accountability and to help each patient reach their tailored nutrition and weight loss goals. The PCIWM program focused on an interdisciplinary approach with the referring physician and other medical professionals for a comprehensive approach to individualized patients’ care plans.

### Data collection

**FIG. 2.**
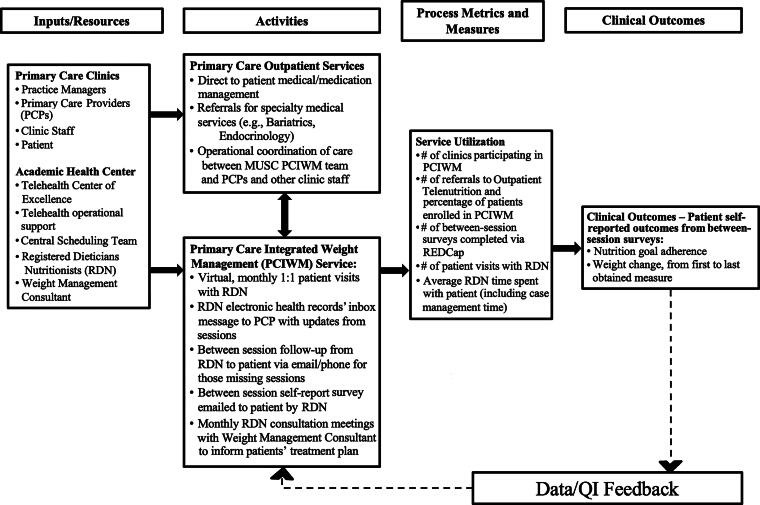
Primary care integrated weight management service logic model.

The pilot PCIWM team aimed to assess process measures as well as initial clinical outcomes (Fig. 2) among patients receiving services since program inception, December 2022 through March 2024. Electronic health records data, via Epic, was exported to obtain demographic, process measures and clinical outcomes data for weight change. For the weight change analysis, 49 of 61 available baseline weight measures were obtained from the medical record chart export and 12 baseline weight measures were obtained from manual chart review. All 3-month follow-up weight measures were collected via manual chart review. In November 2023, the weight management consultant and RDNs internally developed a brief survey, emailed to patients by the RDNs as an additional clinician–patient touchpoint between monthly sessions (Appendix A). Survey data collected between November 2023 and March 2024 were included in this study to assess patient-reported nutrition goal adherence as a measure of behavior change.

Multiple data sources were used to obtain process metrics, initial clinical outcomes, and impacts. Data sources included chart abstraction from electronic health records to obtain patient demographics and weight measures (in kilograms). Program utilization data were maintained by both the RDNs and Outreach Coordinator using Microsoft Excel. This registry was updated weekly by the study team staff. Variables included the overall number of referrals to Outpatient Teleconsultation for Nutrition Services at the large statewide health system, the number of primary care clinics providing referrals to PCIWM, the number of patients enrolled in the PCIWM pilot program, the number of sessions completed with the RDNs (both intake assessments and returning visits), and the number of case consultations completed by RDNs and the Weight Management Consultants.

Descriptive statistics, including measures of central tendency, were used to examine patient characteristics, PCIWM engagement, and outcomes from referral to 3-month follow-up. Data were analyzed using R, v.4.3.0.^34^

## Results

### Program engagement and outcomes

Sixty-one patients were referred to PCIWM from December 2022 through March 2024. Overall, the mean patient age was 46.8 years (SD = 12.5). Additionally, 85.3% of patients identified as female and the majority of patients identified as White (65.5%). Patients also identified as Black (26.2%) and other/unknown (8.2%) racial and/or ethnic groups. Of those referred to PCIWM, 95.1% identified as not Hispanic. The majority of patients were married (63.0%), working full-time (42.6%) and lived in five different rural counties in SC. The mean weight among those enrolled PCIWM was 115.0 kg (SD = 32.0) at baseline.

Of the 61 patients who enrolled, 45.9% cancelled/did not attend their first appointment and did not reschedule, and 8.2% overall did not have updated weights from referral. Therefore, 29% of patients (*n* = 18) were enrolled and actively engaged, 20% of (*n =* 12) patients were enrolled and considered inactive, and 51% of patients (*n* = 31) withdrew from the program during this study period. Of note, patients withdrew for a variety of reasons, including issues related to co-pay requirements, inactivity for more than 3 months, lack of interest in the program, and/or escalation to a higher level of care.

Patients who participated in PCIWM demonstrated promising results. On average, patients had 3.4 RDN visits and spent a mean of 100.7 min with RDNs. On average, 1.5 contact attempts were made to re-engage inactive patients. Of those who attended their follow-ups and had updated weights, 51.9% maintained (within 1 kg) or lost weight (*n* = 14 of 27). Further, 40.7% (*n* = 11) of active patients demonstrated “clinically significant weight loss”—documented as 2–10% of a patient’s body weight—within 3 months.^35^ More specifically, 22.2% of active patients (*n =* 6) demonstrated minimal weight loss for cardiovascular benefits at 2 to <5% and 18.5% of active patients (*n* = 5) demonstrated modest weight loss from 5% to 10% within 3 months.

In terms of BMI, patients had a mean baseline BMI of 41.82 (SD = 11.4, [22.25, 78.85]). Further, active patients demonstrated a mean BMI reduction of 2.69% (SD = 8.8%, [−25.9%, 14.1%]). A majority of active patients (59.3%, *n* = 16) demonstrated a reduction in BMI over the study period. Of those who demonstrated a reduction in BMI, patients demonstrated a mean BMI reduction of 7.92% (SD = 6.7, [−25.94, −1.22]).

Additionally, a total of 44 between-session surveys were sent by the RDNs to active and inactive participants, with a 25% response rate (*N* = 11). Of respondents, most patients (*n =* 7, 63.6%) reported they followed their nutrition goals “well” or “very well” the prior week. Over half of respondents (*n* = 6, 54.5%) reported experiencing challenges to adhering to nutrition goals the prior week. Respondents reported barriers to adhering to nutrition goals included: lack of time (*n* = 2), not feeling motivated to follow their goals (*n* = 1), and “other” challenges (*n* = 3), specifically “travel,” “the holidays,” and “life getting in the way.”

## Discussion

While previous research highlights the role of virtual weight management in increased access to specialty health care services, this study’s findings emphasize the need for active patient engagement within behavioral health interventions, particularly related to weight management.^19,26,27^ Overall, our results indicate that active engagement in virtual nutrition counseling with an RDN may result in favorable weight management outcomes for rural patients in the context of this pilot program. Recent clinical practice guidelines call for more individualized weight loss goals for patients with obesity, and for broadly measuring patient goal attainment beyond weight loss alone.^36^ Therefore, through enhanced coordination and inclusion of PCPs in the PCIWM model, patients may experience more tailored opportunities to set personalized goals, identify strategies to meet their lifestyle behavior goals, and demonstrate greater sustainability of behavior change than a traditional, “one-size-fits-all” approach.”^37^ Rather, a personalized approach to weight management is multi-faceted, involving genetic and environmental influences, clinical characteristics, and health behaviors, attitudes, and perceptions to suggested weight change—all of which influence patient’s engagement in programs such as PCIWM.^9,37^ The PCIWM model shows promise for addressing the new standards of care as 29% of patients were actively engaged, about 64% reported following their nutrition plan, and over half lost or maintained their weight. Further, 40.7% (*n* = 11) of active patients in this sample demonstrated clinically meaningful weight loss within 3 months, resulting in reduced cardiovascular risk factors and improvements in blood sugar/glucose, blood pressure, or cholesterol.^30^ In addition, a majority of active patients (59.3%, *n* = 16) demonstrated a significant reduction in BMI over the study period (M = 7.92%, SD = 6.7%). With such results, this model has the potential to address known barriers to providing obesity treatment in primary care (e.g., PCP lack of time, knowledge and training, increased coordination within interdisciplinary team), and may particularly benefit those who are actively engaged in the program and demonstrate preparedness for lifestyle behavior change. As standards of care evolve, the PCIWM program may also be particularly useful in allowing PCPs to prescribe anti-obesity medications after appropriate lifestyle change interventions have been applied in parallel.

Further, the advent of effective pharmacotherapy will enhance the role of the PCP in weight management, though it is likely that evolving best practices and payment regulations will incentivize or require nutritional counseling and documented patient engagement.^38^ A CoCM approach, augmented with medication prescribing advice, would be particular effective in supporting weight management among patients in a primary care setting.^30,38^ Collectively, integrated RD support may allow PCPs to prescribe medications after appropriate lifestyle interventions that have been applied in parallel.

Lastly, while patient engagement is a critical clinical outcome for success in programs such as PCIWM, low volume uptake persists as a significant challenge for this program. Unfortunately, low uptake and adherence to weight management interventions is well-documented.^39,40^ Echoing challenges with patient engagement among other weight management and CoCM programs focused on behavioral health, this PCIWM may consider additional screening processes to measure patient’s readiness for behavior change prior to PCP referral and subsequent enrollment with RD support. As PCIWM enrollment continues, further research is needed to examine predictors of patient engagement for a tailored approach to weight management and treatment within PCIWM.

### Strengths and limitations

To our knowledge, this was the first program to adapt the CoCM for the purpose of managing adult nutrition and obesity in primary care. Several challenges were observed during this initial phase of the PCIWM pilot program including an overall small sample size and low patient engagement, as 29% of patients were enrolled and actively engaged, and there was a 25% patient response rate to surveys. Additionally, one patient was enrolled as an exception (BMI = 22.25) due to patient-reported and provider concerns of consistent weight gain. Evaluating patient readiness to engage in behavior change may be an important first step to minimize patient withdraw rates due to lack of engagement and/or interest.

Others have also noted limitations regarding patient adherence and challenges measuring behavioral changes during lifestyle change intervention.^41^ Of note, there were no data to provide detailed findings of barriers from referral-to-service connection in this study, or to explain barriers to uptake among referred patients. This may have been influenced by using an external department within the health system for central scheduling from referral to first RD session. While outsourcing scheduling provided support to the clinical team and minimize clinical burden, there were no data to understand why a patient may choose not to start the program following referral. Future study of this PCIWM model should examine feedback from stakeholders including the PCPs, patients, RDNs, and weight management consults to optimize the PCIWM workflow process, increase patient engagement, and promote sustainability of the model.

### Implications for policy and practice

Based on this study’s findings, this clinical and research team have investigated opportunities to improve patient engagement, including assessing patients’ readiness for lifestyle behavior change as is theorized in the Health Belief Model of Behavior Change.^42^ Previous studies have used the Health Belief Model to conceptualize patients’ readiness to improve lifestyle behaviors related to nutrition and physical activity.^42^ Therefore, PCIWM may demonstrate improved adoption and sustainability with the development and implementation of such screening at referring clinical sites.

In addition, the changing landscape in weight management via clinical approaches and policy are important when considering PCIWM’s sustainability.^12,43,44^ Research suggests lifestyle behavior change should remain the first line of defense for weight management.^42,45,46^ Further, recent clinical trials demonstrate improvements in nutrition knowledge, physical activity levels, and weight loss in rural populations, with telehealth interventions showing particular promise.^47^ However, current policy offers limited RDN Telehealth Reimbursement Coverage, which may inhibit the sustainability of PCIWM and similar weight management programs in rural states, such as SC. Ongoing research and advocacy efforts that support the use of virtual weight management programs, particularly among high-risk, rural patients, are critical to the sustainability of such interventions.

Lastly, data extraction from real-world settings—such as PCIWM—often results in missing data. Adaptations to electronic health records may support comprehensive evaluation with program implementation and ongoing adaptations.

## Conclusions

Among engaged patients, PCIWM demonstrated promising, preliminary results to support weight management. However, research is needed to consider patient readiness for behavior change—such as through the Health Belief Model of Behavior Change^42^ or Stages of Change Theory^48^—to encourage improved uptake and sustained participation in dietary counseling for weight management. Additional screening may inform the referral process for greater adherence to PCIWM, partially addressing loss to follow-up and subsequent data challenges in this pilot phase. With a larger sample, research is needed to examine long-term patient outcomes associated with PCIWM engagement, compared with a control group. Lastly, policy barriers, including limited telehealth reimbursement coverage for RDNs, remain relevant in the sustainability and expansion of this model.

## Appendix

### Appendix A: RedCap Survey Distributed to PCIWM Participants

1. What is your name? (First name, Last name)
2. How well do you think you followed your nutrition goals during the past 7 days? (Likert scale: 1, Very well | 2, Well | 3, Not well | 4, I don’t know/remember my diet/nutrition goals)
3. Did you have problems sticking to your nutrition goals over the past 7 days? (1, Yes | 2, No)
4. Please select all of the problems you encountered while trying to follow your nutrition goals over the past 7 days: (1, Goals were too hard to follow | 2, Did not understand my goals | 3, Lack of time | 4, Not enough money to buy food | 5, Not able to travel to buy food | 6, Not motivated to follow my goals | 7, Other (please describe)
5. Please describe the other problems you encountered while trying to follow your nutrition goals. (Open text)
6. How many minutes of physical activity did you get during the past 7 days? Activities like walking, chair exercises, gardening, riding a bike, or something similar can count. (Open text)
7. What was your weight (in pounds) the last time you weighed yourself? (Open text)

## Abbreviations Used

BMI: Body mass index
CoCM: Collaborative care management
PCIWM: Primary Care Integrated Weight Management program
PCP: Primary Care Provider
RDN: Registered Dietitian Nutritionist
SC: South Carolina
